# A Telehealth Privacy and Security Self-Assessment Questionnaire for Telehealth Providers: Development and Validation

**DOI:** 10.5195/ijt.2019.6276

**Published:** 2019-06-12

**Authors:** LEMING ZHOU, ROBERT THIERET, VALERIE WATZLAF, DILHARI DEALMEIDA, BAMBANG PARMANTO

**Affiliations:** 1 DEPARTMENT OF HEALTH INFORMATION MANAGEMENT, SCHOOL OF HEALTH AND REHABILITATION SCIENCES, UNIVERSITY OF PITTSBURGH, PITTSBURGH, PA, USA

**Keywords:** Privacy, Questionnaire Development, Reliability, Security

## Abstract

**Background:**

Telehealth is a great approach for providing high quality health care services to people who cannot easily access these services in person. However, because of frequently reported health data breaches, many people may hesitate to use telehealth-based health care services. It is necessary for telehealth care providers to demonstrate that they have taken sufficient actions to protect their patients’ data security and privacy. The government provided a HIPAA audit protocol that is highly useful for internal security and privacy auditing on health care systems, however, this protocol includes extensive details that are not always specific to telehealth and therefore is difficult to be used by telehealth practitioners.

**Objective:**

The goal of this study was to develop and validate a telehealth privacy and security self-assessment questionnaire for telehealth providers.

**Methods:**

In our previous work, we performed a systematic review on the security and privacy protection offered in various telehealth systems. The results from this systematic review and the HIPAA audit protocol were used to guide the development of the self-assessment questionnaire. The draft of the questionnaire was created by the research team and distributed to a group of telehealth providers for evaluating the relevance and clarity of each statement in the draft. The questionnaire was adjusted and finalized according to the collected feedback and face-to-face discussions by the research team. A website was created to distribute the questionnaire and manage the answers from study participants. A psychometric analysis was performed to evaluate the reliability of the questionnaire.

**Results:**

There were 84 statements in the draft questionnaire. Five telehealth providers provided their feedback to the statements in this draft. They indicated that a number of these statements were either redundant or beyond the capacity of telehealth care practitioners, who typically do not have formal training in information security. They also pointed out that the wording of some statements needed to be adjusted. The final released version of the questionnaire had 49 statements. In total, 31 telehealth providers across the nation participated in the study by answering all the statements in this questionnaire. The psychometric analysis indicated that the reliability of this questionnaire was high.

**Conclusion:**

With the availability of this self-assessment questionnaire, telehealth providers can perform a quick self-assessment on their telehealth systems. The assessment results may be used to identify possible vulnerabilities in telehealth systems and practice or demonstrate to patients the sufficient security and privacy protection to patients’ data.

According to the US Census Bureau, roughly 20% of the US population live in rural areas (U.S. Census Bureau, 2016); however, less than 8% of the nation’s physicians are practicing in rural areas. The majority of these physicians are in primary care such as family practice and general internal medicine (Health Resources and Services Administration, 2014; Jones, Parker, Ahearn, Mishra, & Variyam, 2009). Therefore, it is hard for rural residents to access highly qualified specialists for various types of rehabilitation services (Davidsson & Sodergard, 2016; Iezzoni, Killeen, & O’Day, 2006; Jones et al., 2009). Traveling to major cities for the desired intervention costs a great deal in terms of money and time, which can be a heavy burden for patients and their family members.

Telehealth may be a viable approach for the delivery of high quality health care services to people in rural areas (Cherry et al., 2017) because of the high penetration of broadband connection at home and high ownership of smart mobile devices in recent years. A national survey in 2018 indicated that 89% of American adults used the Internet and 58% of rural American homes are connected to the Internet (Pew Research Center, 2018b). In 2018, 77% of Americans own a smartphone. Even in rural areas, the smartphone ownership rate is 65% (Pew Research Center, 2018a). In other words, the improvement of information and communication technologies make it feasible for delivering telehealth services, such as diagnostic evaluations (Georgeadis, Brennan, Barker, & Baron, 2004; Harper, 2003), assessment and therapy (Hall, Boisvert, & Steele, 2013), and teleconsultation (Wade, Wolfe, Brown, & Pestian, 2005), to remote areas. The nature of telehealth makes it possible for everyone to access high quality care, avoid travel time and costs, and increase collaboration among health care providers (Harper, 2003). Previous studies have also indicated that telehealth is a potentially efficient and effective alternative to hospital-based care (Kairy, Lehoux, Vincent, & Visintin, 2009; Kruse, Krowski, et al., 2017).

Despite these benefits, patients have concerns when they are invited to receive health care services via telehealth technologies (e.g., videoconferencing and store-and-forward) instead of in-person clinic visits. One of these concerns is about security and privacy of their health data (Hale & Kvedar, 2014; Hall & McGraw, 2014; He, Naveed, Gunter, & Nahrstedt, 2014).

It is not surprising that these patients have the security and privacy concern because health data breaches have occurred frequently, impacting a growing number of people in recent years. Currently, cyber-attacks targeting medical information has increased 22 percent a year with 112 million compromised records back in 2015 (Kruse, Frederick, Jacobson, & Monticone, 2017).

Protected Health Information (PHI) breaches are also costly to the healthcare industry. According to the Cost of Data Breach Study released by IBM Security and the Ponemon Institute in 2018, the average global cost of a health data breach per lost or stolen record was US $380 (Ponemon Institute & IBM Security, 2018). Overall, the US health care industry spent approximately $67 billion dealing with issues triggered by PHI breaches on activities such as conducting investigations, notifying customers, recovering data, subscribing to credit monitoring services for customers, hiring knowledgeable security personnel, and strengthening the security measures of information technology (IT) systems (Ponemon Institute & IBM Security, 2018).

To reduce the security and privacy concerns from patients, telehealth providers are the ones who provide the tutorial or education to their patients. Therefore, telehealth providers should be ready to do this work. It is also critical for telehealth providers to be familiar with the security features of their telehealth systems and practice so that they can be prepared to mitigate breaches and protect their customer base since quicker security violation identification and containment practices have resulted in lower costs to the organization (Ponemon Institute & IBM Security, 2018). For both purposes, telehealth providers need to perform a self-assessment on their telehealth systems and practice.

The Health Insurance Portability and Accountability Act of 1996 (HIPAA) implemented physical and technical safeguards to protect sensitive information from cyber criminals (Kruse, Krowski, et al., 2017). The physical security addressed in HIPAA include workstation use, device and media controls, and facility access controls. The technical safeguards include unique user identification numbers, emergency access procedures, automatic logoff, encryption and decryption (Kruse, Krowski, et al., 2017). The HIPAA Security Rule has been updated due to the threat of cyber-attacks in health care in recent years.

The HIPAA audit protocol offered by the Office for Civil Rights (OCR) in the Department of Health and Human Services (DHHS) provided extensive details about the security and privacy protection requirements on health IT systems (https://www.hhs.gov/hipaa/for-professionals/compliance-enforcement/audit/protocol/index.html). However, it is very challenging for typical telehealth providers to use this protocol to perform a security and privacy self-assessment on their telehealth systems and practice. The HIPAA audit protocol was prepared for security and privacy compliance officers, not for telehealth providers who typically have no formal training in information security and privacy.

## PREVIOUS WORK

In our previous study, we performed a systematic review on papers published between 2004 to 2016 to examine telehealth privacy and security practices used by healthcare providers (Watzlaf, Zhou, DeAlmeida, & Hartman, 2017). All 21 studies examined in the systematic review discussed some aspect of privacy and security. For example, the patient’s rights to include informed consent, data accessibility, confidential communications; the patient’s ability to amend their information; how video sessions are retained; authorizations for release of information to other countries, websites, and third parties; accounting of disclosures, purging and/or deletion schedule of files on mobile devices and audio and video muting to maintain privacy; and the technical aspects of security to encompass encryption, two-factor authentication, data backup, storage and recovery to meet HIPAA requirements, National Institute of Standards and Technology (NIST) and Health Level-7 (HL7) recommendations. A relatively weaker aspect of telehealth security determined through our systematic review was physical safeguards. Only eight studies addressed the criticality of having a secure server location, data back-up, and maintaining a secure environment for the telehealth practice.

To improve the practices of the entire telehealth team, we proposed the design and utilization of a telehealth privacy and security self-assessment in the form of a questionnaire that telehealth practitioners can use to easily and conveniently evaluate their telehealth systems and practice.

## OBJECTIVE

The objective of this study was to create a reliable and easy-to-use telehealth security and privacy self-assessment questionnaire for telehealth providers. The questionnaire may be used to evaluate the current status of privacy and security in telehealth systems and practice.

## METHODS

### QUESTIONNAIRE DEVELOPMENT

#### STEP 1: LITERATURE REVIEW

The development of the desired questionnaire was based on the results from our systematic review and the HIPAA audit protocol (Watzlaf et al., 2017). From the systematic review study, it was determined that the best criteria to examine the strength of telehealth programs were policies, storage, consent, transmission/accessibility, encryption, data backup plans, training, authentication /access control, authorization, and secure networks.

#### STEP 2: DRAFT QUESTIONNAIRE CREATION

The research team had weekly in-person meetings for several months to create statements for the questionnaire. This step took a long time because each statement was written by the research team according to the results of the systematic review and the HIPAA audit protocol. There were 84 statements in the first draft of the questionnaire.

#### STEP 3: RELEVANCE AND CLARITY ASSESSMENT

This draft was placed on the Web-based Qualtrics system. Five telehealth providers were invited to evaluate the relevance and clarity of the draft. They were also given an option to provide further comments on each statement. These five telehealth providers were considered as a part of this research team since they directly contributed to the creation of the questionnaire. All five telehealth providers went through these 84 statements to determine their relevance and clarity in terms of the study purpose on a scale of 1 to 4, where 1 means no relevance or clarity, and 4 means high relevance or clarity. If three or more providers rated the relevance of a statement 1 or 2, the statement was removed from the draft. If any one of these telehealth providers rated the clarity of a statement 1 or 2, the wording of the statement was adjusted. The research team had multiple in-person meetings to discuss the feedback and perform changes accordingly. At the end of this step, there were 49 statements in the questionnaire.

#### STEP 4: WEBSITE CREATION AND STUDY DATA COLLECTION

After everyone on the research team agreed on the content validity of the statements in the questionnaire, a website was created to distribute this questionnaire and manage respondents’ answers. The purpose was not only to collect data for this study, but to provide the self-assessment service to telehealth providers in the future. On the website, each user is required to create an account before he/she can provide answers to the statements. All responses to the statements are stored in a secure database and a summary is provided to participants. The website also displays a page that contains links to resources such as security terms, references, and the HIPAA audit protocol.

#### STEP 5: THIRTY ONE (31) TELEHEALTH PROVIDERS PARTICIPATED IN THE STUDY USING THE NEW SELF-ASSESSMENT QUESTIONNAIRE

The participants could select “Yes (0)”, “No (1)”, or “I don’t know (2)” as their response to each statement. Here “yes” means the participant knew the situation and the organization’s telehealth system/practice had that specific security feature; “no” means the participant knew the actual situation but the telehealth system/practice did not have the corresponding security feature; “I don’t know” means the telehealth practitioner was not clear about this particular security feature in the organization’s telehealth system/practice, which typically (not always) also means that the organization’s telehealth system/practice did not have that particular security feature. The obtained data were used to evaluate the reliability and validity of the new questionnaire. During the study, the study participants were also asked to provide answers to a few demographic questions. All of the answers to the questionnaire were collected with the website created by the research team. The details of the study procedure and the data analysis are presented in the following sections.

### STUDY DESIGN

Before we conducted the study, we communicated with the Institutional Review Board (IRB) office at the University of Pittsburgh. The IRB office instructed us that the project we described to them does not require IRB review and approval.

We recruited study participants via email and phone call. Potential study participants (telehealth providers) were identified from the Telemedicine & Telehealth Service Provider Directory created by the Arizona Telemedicine Program (https://telemedicine.arizona.edu/servicedirectory), the American Telemedicine Association website, the California Telehealth Resource Center Specialty Provider List, the North Carolina Telehealth Programs and Service Providers Chart, and the Grantee profiles of 2017 developed by the Office for Advancement of Telehealth. Telehealth providers’ emails and phone numbers were collected from these websites. An email with a brief introduction to the project was sent to these potential study participants. The email recipients can simply click the link in the email to access the questionnaire website and provide their responses to the questionnaire. The potential study participants were followed up via email and phone, with their permission, if they agreed to participate in the study but did not provide their response to the questionnaire one week after accepting the invitation. Study participation was completely voluntary, and participants could stop participating in the study at any time. Additionally, there was no compensation for the participants who completed the questionnaire.

### STATISTICAL ANALYSIS

All statistical analyses were conducted using SPSS version 25 (IBM). The internal consistency of the questionnaire was evaluated using Cronbach’s alpha. For research or evaluation, a value of 0.7 to 0.8 in Cronbach’s alpha is considered reliable. Descriptive statistics were calculated for all the items in the questionnaire.

## RESULTS

The final product was a questionnaire with 49 statements, which were arranged into 10 domains: 8 statements on policies, 6 statements on storage, 6 statements on consent, 1 statement on transmission/accessibility, 3 statements on encryption, 3 statements on data backup plans, 3 statements on training, 5 statements on authentication/access control, 4 statements on authorization, and 10 statements on secure networks.

The participants in this study were 31 telehealth providers who provide telehealth services or contribute to a telehealth operation at a healthcare related organization in the United States. Descriptive characteristics of the organizations that the 31 participants work for and the participants’ demographics were collected. Tables 1 and 2 show the individual and organizational descriptive breakdown.

Based on the responses from the 31 study participants to the 49 statements in the questionnaire, the Cronbach’s alpha was calculated. The obtained value was 0.906. This result means that the statements in the questionnaire are highly consistent and reliable for assessing the privacy and security practices of telehealth. Table 3 shows all 49 statements in the telehealth security self-assessment questionnaire.

In addition to the reliability test, descriptive analysis was performed to determine which areas of the telehealth programs were strong and weak among the organizations of study participants. Table 4 shows the number and percentage of study participants who answered “yes (0)”, “no (1)”, and “I don’t know (2)” to statements in each domain.

First, the answers to statements in the 10 domains of the questionnaire were compared to determine in which areas study participants have capabilities or knowledge, in which areas they are aware that they do not have, and areas where they are uncertain of their capabilities. From Table 4 it is clear that the domain that the participants answered “yes” most frequently was in security and privacy policies. This was followed by authentication/access control, authorization, data backup plans, training, consent, encryption, secure networks, transmission/accessibility, and storage.

Following the analysis at the domain level, the domains with the low percentage of “yes” responses were examined at the statement level. Table 5 shows a summary of the responses to each statement in the self-assessment questionnaire.

The five domains with the lowest percentage of “yes” responses (storage, transmission/accessibility, secure networks, encryption, and consent) were evaluated to determine the statements which contributed to a lower percentage of “yes” responses. Additionally, Authorization and Data backup plans were incorporated in this analysis since they contained high percentages of “I don’t know” responses (18.5% and 22.6%, respectively).

As stated earlier, storage domain had the lowest percentage of “yes” responses (49.5%). When analyzing the specific questions for the storage domain, Q13 and Q14 were areas that had a high percentage of “no” and “I don’t know” responses. Q13 asked participants if they “monitor whether any of the transmitted data during a telehealth session is stored on the patient’s computer or other device’s hard drive.” Of the 31 participants, 19 (61.3%) stated that they do not monitor whether the transmitted data is stored on the patient’s computer or other device’s hard drive. Q14 asked participants if the telehealth system they utilize is “able to trigger remote erase of a mobile device used for telehealth sessions if the mobile device is lost or stolen.” Eleven participants (35.5%) indicated that they are unsure of whether or not their telehealth system contains that capability.

Following storage, transmission/accessibility had the next lowest percentage of “yes” responses at 51.6%. The only statement for this domain asked respondents if PHI generated during the telehealth session is accessible to others outside of the organization (such as law enforcement, government officials, etc.) as long as they have proper authorization. Five respondents (16.1%) were unsure of whether that was the case for their telehealth program. Ten respondents (32.3%) stated that the PHI was not accessible to others outside the organization even if they have proper authorization.

The percentage of “yes” responses to the statements in the secure networks domain was 69.7%. Q41 and Q46 had the highest percentages of “no” answers for this domain. Q41 asked participants if they “use a Virtual Private Network (VPN) to access important websites.” Seven respondents (22.6%) stated that they did not utilize a VPN to access important websites. Q46 asked respondents if “there is a security evaluation conducted by an independent party on the telehealth system to verify features such as authentication, encryption, authorization, Wi-Fi settings, data management plan, and all other proper privacy and security features.” Seven participants (22.6%) answered “no.” Q46 also had the greatest percentage of “I don’t know” answers for the secure network domain (35.0%). Q43 and Q44 also saw a high percentage of participants who reported “I don’t know.” For Q43, 9 participants (29.0%) stated that they were unsure if privacy and security features of mobile applications used in telehealth practice are carefully researched before being downloaded. Similarly, for Q44, 9 participants (29.0%) stated that they did not know if a “disaster recovery plan was in place in for the data collected during telehealth practice sessions” in their respective telehealth programs.

The encryption domain followed secure networks with the next lowest percentage of “yes” responses with 71.0%. Q22 and Q24 had high percentages of respondents reporting “I don’t know.” Specifically, 8 participants (25.8%) reported that they did not know if their “telehealth system vendor included details about encryption algorithms, the key management approach, and what specific data are encrypted.” Also, 11 participants (35.5%) reported that they did not know if encryption keys are periodically updated to meet the privacy and security protocol.

After encryption, the consent domain had the next lowest percentage of “yes” responses at 71.5%. Q17 asked if “the patient informed consent states that telehealth sessions may be recorded and pictures may be taken and stored.” Q16 asked the participant whether “the patient informed consent includes the privacy and security features of the telehealth system.” Q18 asked the participants if “the patient informed consent includes recommendations that the environment and surroundings be secured.” Q17, Q18, and Q16 had high percentages of “no” responses at 45.2%, 29.0%, and 22.6%, respectively. Q20 asked if the participant’s organization “informs the patients of potential security risks when PHI is transferred between the healthcare provider and the telehealth system.” Eight participants (25.8%) answered “I don’t know.”

Although the data backup plans domain was not one of the bottom five domains for “yes” answers by the participants, it had a high percentage of “I don’t know” answers. For Q26, eight participants (25.8%) stated that they did not know if there “is a data backup plan reviewed and updated on a regular basis.” For Q25, seven respondents (22.6%) stated that they did not know “if there was a technology breakdown or a data backup plan” in place in each of their respective telehealth programs. Finally, for Q27, six participants (19.4%) declared that they did not know if there are “appropriate redundant systems in place that ensure the availability of telehealth services when one or a few components of the system is not working.”

Similar to the data backup plans domain, authorization was another domain that was not in the bottom five domains for “yes” answers by the participants. However, they had a high percentage of “I don’t know” answers. For Q37, nine respondents (29.0%) reported that they did not know if “qualified individuals with proper certifications and backgrounds in privacy, security, and HIPAA regulations evaluate all requests for PHI” for their respective telehealth programs. For Q38, seven respondents (22.6%) reported that they did not know if “patients receive an accounting of disclosures upon written request.” Finally, for Q39, six participants (19.4%) documented that they did not know if “a patient’s request for a restriction of uses and disclosures of PHI that is generated from the telehealth system is honored.

Table 6 summarizes the identified privacy and security vulnerabilities from the 31 participants’ responses to the self-assessment questionnaire. They may guide telehealth providers to enhance the privacy and security of their telehealth systems and practice.

## DISCUSSION

### PRINCIPAL FINDINGS

To assist telehealth providers to perform security and privacy self-assessment on their telehealth systems and practice so that they can provide education to their patients and also be prepared if a health data breach occurs, we created a telehealth security self-assessment questionnaire. The study result indicated that this questionnaire is highly reliable.

The responses from 31 study participants were summarized, which indicated the areas the organizations of these study participants did well on in terms of security of their telehealth systems and practice, and the vulnerabilities in some areas. The individual report for each study participant may also be used to guide the corresponding organization to improve the security of their telehealth systems and practice.

We encourage more telehealth providers to visit the website and provide their answers to the 49 statements. If needed, they can make changes in their telehealth systems and practice according to the identified vulnerabilities. These telehealth providers can answer this telehealth security self-assessment questionnaire and view the history of their answers to determine which areas they have improved, and which areas still need further work.

Please note, the reported summary was from 31 organizations. The data should be monitored to observe if the five domains that had the lowest percentage of “yes” responses (storage, transmission/accessibility, secure networks, encryption, and consent) persist when a greater sample of participants/organizations is utilized. Additionally, the domains that received high rates of “I don’t know” responses should be monitored to determine where education opportunities can be applied.

### LIMITATIONS

There were limitations to this study. First, there were 31 participants/organizations that completed the telehealth security self-assessment questionnaire. Because of the sample size we have at this moment, it is not meaningful to perform further statistical analysis, for instance, the difference among telehealth providers’ answers to these statements in terms of the type of their institutions, size of the telehealth institute, size of their IT team, size of security and privacy team, and year of using telehealth. The current sample size is also not sufficient for performing an exploratory factor analysis to determine the constructs in this questionnaire and whether these constructs are consistent with the domains we determined when the questionnaire was created. When the number of participants is significantly larger, further statistical analysis will be conducted to determine the relationship between the participants’ characteristics, organizations’ characteristics, and their answers to the questionnaire. It will also be possible to conduct an exploratory factor analysis to determine the constructs in this questionnaire and whether they are consistent with the domains assigned in this study.

Second, since federal regulations such as HIPAA were applied in the questionnaire, we restricted the study to individuals and organizations that practice telehealth in the United States. The European Union (EU) data privacy rule, General Data Protection Regulation (GDPR), is a new regulation in EU law that protects the data privacy for people in the EU and the European Economic Area (EEA) (European Union, 2018). According to GDPR, US healthcare organizations must take measures if they process personal data of EU individuals, if the organization is established in the EU, if the organization is established outside of the EU and processes data for goods and services offered in the EU, and if the organization monitors the behavior of EU individuals. Thus, with regard to telehealth services, healthcare organizations should conduct security assessments to determine if their security infrastructure is in compliance with GDPR standards. Additionally, external environmental security assessments should be conducted to confirm if their current or potential vendors are GDPR compliant (Meinert et al., 2018). Research on privacy and security practices in relation to GDPR compliance may be effective in helping healthcare organizations when providing telehealth services.

## CONCLUSION

In summary, this telehealth self-assessment questionnaire proved to be internally reliable. The questionnaire created and evaluated in this study provides a reliable means for telehealth providers and professionals to self-assess their telehealth systems and programs. When there are more telehealth providers answering the questionnaire, the obtained data may be used to examine the strengths and weaknesses of various programs’ privacy and security issues in their telehealth systems and practice. The results will reflect the current security and privacy situation in telehealth practice in the US and guide health care organizations to improve the security of their telehealth systems and practice.

## Figures and Tables

**Table 1:** Demographic Information of Study Participants (N = 31)

Characteristics	n	%
**Gender**		
Male	17	54.8
Female	14	45.2
**Age**		
18–25	1	3.2
26–35	2	6.5
36–45	6	19.4
46–55	7	22.6
56–65	13	41.9
≥ 66	2	6.5
**Background**		
Business Administration	6	19.4
Health Care Administration	3	9.7
Health Science	4	12.9
Information Technology	3	9.7
Nursing	3	9.7
Public Health	2	6.5
Other	10	32.3
**Position**		
Chief Executive Officer	6	19.4
Chief Operating Office	1	3.2
Information Technology Specialist	1	3.2
Physician	3	9.7
Program Manager	4	12.9
Psychiatrist/Psychologist	2	6.5
Social worker	1	3.2
Telehealth supervisor	2	6.5
Other	11	35.5
**Years of Work Experience in Security and Privacy**		
0–2	9	29.0
3–5	4	12.9
6–10	3	9.7
11–15	2	6.5
16–25	9	29.0
≥26	4	12.9

**Table 2:** Summary of Participants’ Organization Information

Organization Characteristics	n	%
**Type**		
Educational Institute	4	12.9
Hospital	6	19.4
Hospital Network	3	9.7
Independent Practice Association	2	6.5
Managed Care Organization	1	3.2
Physician Group Practice	4	12.9
Other	11	35.5
**Years of Using Telehealth**		
1–3	6	19.4
4–6	9	29.0
7–10	4	12.9
>10	12	38.7
**Size of IT Team**		
0	2	6.5
1–3	11	35.5
4–10	8	25.8
11–20	2	6.5
21–50	2	6.5
>50	6	19.4
**Number of IT Security Personnel**		
0	3	9.7
1–3	12	38.7
4–10	7	22.6
11–20	3	9.7
21–50	2	6.5
>50	4	12.9

**Table 3:** Statements of the Telehealth Security Self-Assessment Questionnaire

D1: Policies
Q1. Does the telehealth system (vendor) have privacy policies in place? Q2. Does the telehealth system (vendor) have security policies in place? Q3. Are the privacy and security policies easy to understand? Q4. Do the telehealth privacy and security policies include guidance on the best method to use to protect the security of patient information? Q5. Are business associate agreements (BAAs) in place between the telehealth system (vendor) and other entities that do business with the telehealth system (vendor)? Q6. If the vendor shares Protected Health Information (PHI) from the telehealth system (vendor) to other entities, are the privacy and security policies of those other entities checked before sharing? Q7. Are the privacy and security policies and procedures kept current to meet federal and multi-state regulations? Q8. Do the privacy and security features that are part of the telehealth system (vendor) meet federal and multi-state regulations?
**D2. Storage**
Q9. Will PHI generated between the provider and patient be stored in any capacity by the telehealth system (vendor)? Q10. Does the telehealth system (vendor) include guidance and information to clients on how best to store PHI which may include recordings of telehealth sessions? Q11. When considering cloud service for data storage, is the telehealth system (vendor) compliant in keeping PHI highly secure? Q12. Are clients discouraged from storing patient related information generated during the telehealth session offline on other storage devices? Q13. Do you monitor whether any of the transmitted data during a telehealth session is stored on the patient’s computer or other device’s hard drive? Q14. Is the telehealth system able to trigger remote erase of a mobile device used for telehealth sessions, if the mobile device is lost or stolen?
**D3. Consent**
Q15. Is the patient’s or representative’s informed consent obtained before the telehealth session begins? Q16. Does the patient informed consent include the privacy and security features of the telehealth system? Q17. Does the patient informed consent state that telehealth sessions may be recorded and pictures taken and stored? Q18. Does the patient informed consent include recommendations that the environment and surroundings be secure? Q19. Are patients provided the right to authorize a transfer of PHI outside of the existing system (e.g., to a biller, 3rd party payer, other entity)? Q20. Are patients informed of the potential security risks when PHI is transferred between the health care provider and the telehealth system (vendor)?
**D4. Transmission/Accessibility**
Q21. Is PHI generated during the telehealth session accessible to others outside of the organization (such as law enforcement, government officials, etc.) as long as they have proper authorization?
**D5. Encryption**
Q22. Does the telehealth system (vendor) include details about encryption algorithms (such as the length of the key, for example, AES-256, the key management approach, and what specific data are encrypted)? Q23. Do the encryption methods meet recognized standards from HIPAA, HITECH, the International Standards Organization (ISO) and the National Institute of Standards and Technology (NIST) as well as multi-state regulations? Q24. Are encryption keys periodically updated to meet the privacy and security policy?
**D6. Data backup plan**
Q25. If there was a technology breakdown, is there a data backup plan (e.g., be able to create and maintain exact copies of ePHI, establish what ePHI should be backed up, such as telehealth sessions/data) in place? Q26. Is the data backup plan reviewed and updated on a regular basis (at least yearly)? Q27. Are there appropriate redundant systems in place that ensure the availability of telehealth services even when one or a few components of the system are not working?
**D7. Training**
Q28. Is employee training provided on computer network privacy and security AND mobile device privacy and security? Q29. Is HIPAA training, which includes instructional material tailored for telehealth privacy and security, provided at least on an annual basis, for all staff that use the telehealth system? Q30. Are the risks of social media connections (e.g. risks of inadvertent linking of patients via social media as a result of using mobile devices with downloaded social media accounts on the device) discussed with all users of the telehealth system?
**D8. Authentication/Access Control**
Q31. Is proper user authentication (username, passwords, fingerprinting, PINs, and security questions) established before logging into the telehealth session? Q32. Do you use strong passwords (uppercase, lowercase, minimum length, special symbols, digits, etc.) to access the telehealth system? Q33. Is there an inactivity time out function available on the telehealth system that requires re-authentication to access the system after the timeout period has ended? Q34. Is unauthorized viewing of patient information prevented by applying access controls (e.g., role-based, user-based, context-based access controls)? Q35. Are all of the smart devices (smartphones, tablets, smartwatch etc.) that are used in telehealth sessions, password protected and encrypted?
**D9. Authorization**
Q36. Is prior written patient authorization required before any PHI content, developed as part of the telehealth session, is shared with other requestors? Q37. Do qualified individuals with proper certification and backgrounds in privacy, security, and HIPAA regulations evaluate all requests for PHI? Q38. Do patients receive an accounting of disclosures upon written request?Q39. Will a patient’s request for a restriction of uses and disclosures of PHI that is generated from the telehealth system be honored?
**D10. Secure Networks**
Q40. Do you connect only to secure networks (e.g., HTTPS, VPN, TLS, SSL) when using telehealth systems and avoid unsecure networks (e.g., public Wi-Fi)? Q41. Do you use a Virtual Private Network (VPN) to access important websites? Q42. Do you use Wi-Fi Protected Access-2 (WPA2) certification with AES-256 encryption for Wi-Fi? Q43. Are privacy and security features of mobile apps used in telehealth practice carefully researched before being downloaded? Q44. Is a disaster recovery plan (e.g., procedures in place to restore lost data, the types of data to be restored and copy of the disaster plan is readily available when needed) in place for the data collected during telehealth practice sessions? Q45. Is an incident response plan in place for your telehealth practice? Q46. Is there a security evaluation conducted by an independent party on the telehealth system to verify features such as Authentication, Encryption, Authorization, Wi-Fi settings, Data Management Plan, and all other proper privacy and security features? Q47. Do you verify the source and integrity of the data when sending or receiving data during the telehealth session? Q48. Are audit trails (a feature that records user activity in a telehealth system/vendor) used to track who has access to PHI that is collected during the telehealth session? Q49. Are there up-to-date anti-virus, anti-malware programs installed on all devices used for telehealth sessions?

**Table 4:** Summary of Responses to Statements in Each Domain. The Domains Are Sorted by Percentage of Participants Who Answered “Yes”

Domains	n (%)		
	Yes (0)	No (1)	I don’t know (2)
**D1. Polices**	232 (93.5)	7 (2.8)	9 (3.6)
**D8. Authentication/Access Control**	137 (88.4)	7 (4.5)	11 (7.1)
**D9. Authorization**	96 (77.4)	5 (4.0)	23 (18.5)
**D6. Data backup plans**	69 (74.2)	3 (3.2)	21 (22.6)
**D7. Training**	68 (73.1)	15 (16.1)	10 (10.8)
**D3. Consent**	133 (71.5)	38 (20.4)	15 (8.1%)
**D5. Encryption**	66 (71.0)	3 (3.2)	24 (25.8)
**D10. Secure Networks**	216 (69.7)	34 (11.0)	60 (19.4)
**D4. Transmission/Accessibility**	16 (51.6)	10 (32.3)	5 (16.1)
**D2. Storage**	92 (49.5)	56 (30.1)	38 (20.4)

**Table 5:** Descriptive Analysis of the Responses to the Telehealth Security and Privacy Self-assessment Questionnaire (N=31)

Domains	Statements	n (%)		
		Yes (0)	No (1)	I don’t know (2)
**D1. Polices**	Q1	31 (100)	0 (0)	0 (0)
	Q2	31 (100)	0 (0)	0 (0)
	Q3	27 (87.1)	2 (6.5)	2 (6.5)
	Q4	26 (83.9)	4 (12.9)	1 (3.2)
	Q5	30 (96.8)	0 (0)	1 (3.2)
	Q6	26 (83.9)	1 (3.2)	4 (12.9)
	Q7	30 (96.8)	0 (0)	1 (3.2)
	Q8	31 (100)	0 (0)	0 (0)
**D2. Storage**	Q9	17 (54.8)	11 (35.5)	3 (9.7)
	Q10	13 (41.9)	9 (29.0)	9 (29.0)
	Q11	24 (77.4)	0 (0)	7 (22.6)
	Q12	18 (58.1)	8 (25.8)	5 (16.1)
	Q13	9 (29.0)	19 (61.3)	3 (9.7)
	Q14	11 (35.5)	9 (29.0)	11 (35.5)
**D3. Consent**	Q15	28 (90.3)	2 (6.5)	1 (3.2)
	Q16	21 (67.7)	7 (22.6)	3 (9.7)
	Q17	17 (54.8)	14 (45.2)	0 (0)
	Q18	21 (67.7)	9 (29.0)	1 (3.2)
	Q19	27 (87.1)	2 (6.5)	2 (6.5)
	Q20	19 (61.3)	4 (12.9)	8 (25.8)
**D4. Transmission/Accessibility**	Q21	16 (51.6)	10 (32.3)	5 (16.1)
**D5. Encryption**	Q22	21 (67.7)	2 (6.5)	8 (25.8)
	Q23	26 (83.9)	0 (0)	5 (16.1)
	Q24	19 (61.3)	1 (3.2)	11 (35.5)
**D6. Data backup plans**	Q25	24 (77.4)	0 (0)	7 (22.6)
	Q26	22 (71.0)	1 (3.2)	8 (25.8)
	Q27	23 (74.2)	2 (6.5)	6 (19.4)
**D7. Training**	Q28	29 (93.5)	2 (6.5)	0 (0)
	Q29	24 (77.4)	4 (12.9)	3 (9.7)
	Q30	15 (48.4)	9 (29.0)	7 (22.6)
**D8. Authentication/Access Control**	Q31	28 (90.3)	2 (6.5)	1 (3.2)
	Q32	28 (90.3)	3 (9.7)	0 (0)
	Q33	28 (90.3)	1 (3.2)	2 (6.5)
	Q34	26 (83.9)	0 (0)	5 (16.1)
	Q35	27 (87.1)	1 (3.2)	3 (9.7)
**D9. Authorization**	Q36	28 (90.3)	2 (6.5)	1 (3.2)
	Q37	20 (64.5)	2 (6.5)	9 (29.0)
	Q38	23 (74.2)	1 (3.2)	7 (22.6)
	Q39	25 (80.6)	0 (0)	6 (19.4)
**D10. Secure Networks**	Q40	27 (87.1)	3 (9.7)	1 (3.2)
	Q41	22 (71.0)	7 (22.6)	2 (6.5)
	Q42	24 (77.4)	1 (3.2)	6 (19.4)
	Q43	21 (67.7)	1 (3.2)	9 (29.0)
	Q44	20 (64.5)	2 (6.5)	9 (29.0)
	Q45	19 (61.3)	6 (19.4)	6 (19.4)
	Q46	13 (41.9)	7 (22.6)	11 (35.5)
	Q47	20 (64.5)	3 (9.7)	8 (25.8)
	Q48	22 (71.0)	2 (6.5)	7 (22.6)
	Q49	28 (90.3)	2 (6.5)	1 (3.2)

**Table 6:** Telehealth Privacy and Security Vulnerabilities Examined by the Telehealth Privacy and Security Self-assessment Questionnaire

Domain	Vulnerabilities
**Storage**	Lack of monitoring if transmitted data during a telehealth session is stored on the patient’s computer or other device’s hard drive.
**Transmission / Accessibility**	Unsure of whether or not PHI generated during the telehealth sessions is accessible to others outside of the organization who have proper authorization.
**Secure networks**	Did not always utilize a VPN to access important websites.
	Lacked mobile application security research before downloading and were unsure about having a disaster recovery plan and security evaluation.
**Encryption**	Did not always know if their telehealth system vendor included details about encryption algorithms.
	Did not always know if encryption keys are periodically updated to meet their privacy and security protocol.
**Consent**	The patient informed consent did not always include that telehealth sessions may be recorded and pictures may be taken and stored; the privacy and security features of the telehealth system; and did not address environment and surrounding security recommendations.
	Did not always know whether or not the organization provided the patients with information pertaining to the security risks of information transfer between the organization and the telehealth system vendor.
**Data backup plans**	Lack of knowledge on whether the data backup plan was reviewed and updated on a regular basis (at least yearly) with a technology breakdown for the telehealth program.
	Lack of knowledge on whether appropriate redundant systems are in place for their telehealth system.
**Authorization**	Not always certain if there were certified privacy and security professionals to evaluate requests for PHI from the telehealth sessions.
	Not always certain if patients receive an accounting of disclosures upon written request.
	Not always aware if a patient’s request for a restriction of users and disclosures of PHI that is generated from the telehealth system is honored.

## ABBREVIATIONS

BAA: Business Associate Agreement
DHHS: Department of Health and Human Services
EEA: European Economic Area
EU: European Union
GDPR: General Data Protection Regulation
HIPAA: Health Insurance Portability and Accountability Act
HL7: Health Level-7
IRB: Institutional review board
NIDILRR: National Institute on Disability, Independent Living, and Rehabilitation Research
NIST: National Institute of Standards and Technology
NSF: National Science Foundation
OCR: Office for Civil Rights
PHI: Protected Health Information
VPN: Virtual Private Network
WPA2: Wi-Fi Protected Access-2
